# Evaluation of relationship between memory and temperament in 18-28 years old students

**DOI:** 10.22088/cjim.15.2.334

**Published:** 2024

**Authors:** Reihaneh Moeini, Maliheh Mohammadi Sagh, Mahbobeh Faramarzi, Payam Saadat, Morteza Mojahedi, Narjes Gorji, Alijan Ahmadi Ahangar

**Affiliations:** 1 Traditional Medicine and History of Medical Sciences Research Center, Health Research Institute, Babol University of Medical Sciences, Babol, Iran; 2 Student Research Committee, Babol University of Medical Sciences, Babol, Iran; 3 Population and Family Spiritual Health Research Center, Health Research Institute, Babol University of Medical Science, Babol, Iran; 4 Mobility Impairment Research Center, Health Research Institute, Clinical Research Development Unit of Rouhani Hospital , Babol University of Medical Sciences, Babol, Iran

**Keywords:** Mizaj, Temperament, Individualized medicine, Wechsler memory scale, Persian medicine.

## Abstract

**Background:**

Mizaj (Temperament) is a concept to express individual differences in Persian medicine and according to this theory, there is a relationship between Mizaj type and the abilities of different body organs. This cross-sectional study aimed to investigate the relationship between the type of Mizaj and the memory score (Quotient).

**Methods:**

The target population was the 18 to 38 years old students of Babol University of Medical Sciences. Mojahedi’s Mizaj questionnaire (MMQ) was used for determining the whole Mizaj. The physical Persian version of Wechsler Memory Scale III (WMS III) was used to assess memory score. The collected data were analyzed by SPSS Version 22 and the chi square (x2) and t-test were run and p- value 0.05 was considered as significant difference.

**Results:**

Forty-two of participants were females and 18 were males. The average age of them was 23.6 (21-27). The average of Memory Quotient (MQ) was 122.1 ± 5.7. The average of MQ in warm Mizaj was 125.46 ± 1.2 and in cold Mizaj was 118.79 ± 6.5. The difference between two groups is statistically significant (p< 0.001). The average of MQ in dry Mizaj was 124.16 ± 2.67 and in wet Mizaj was 118.40 ± 7.64. The difference between two groups is statistically significant (P= 0.005).

**Conclusion:**

The results showed there are significant relationship between memory score and warm/cold Mizaj and dry /wet Mizaj. It means students with warm or dry Mizaj had better memory score than students with cold or wet Mizaj. This relation was also detected between subtypes of memory and Mizaj expect between working memory and dry/wet Mizaj. These results are in accordance with theories in PM which indicate people with warm Mizaj and dry Mizaj have better memory and people with cold Mizaj and wet Mizaj have weaker memory and are more at risk of memory dysfunction.

Paying attention to individual differences in medicine has increased significantly in the last two decades and is referred to as Individualized Medicine (1). That is while, this issue has been one of the basic principles in traditional medicines, such as Persian medicine (PM) for more than one thousand years. Individual differences in PM are categorized under the concept of Mizaj (temperament) (2). Mizaj is defined as the individual manifestations and characteristics caused by the difference in the constituents of people's bodies (3). This concept is one of the most important foundations of PM based on individualized approach which has a special role in determining the type of dietary regimen, proper medications and lifestyle recommendations of individuals (4).

This person-center view divides people based on their biological, physical and psychological characteristics into 9 main categories include four singular Mizajes (warm, cold, wet, and dry) and four compound Mizajes (warm and wet, warm and dry, cold and wet, cold and dry) and one temperate Mizaj (2).

Mizaj identification in classic method is performed based on 10 qualitative main criteria including state of the skin (touch), hair, soft tissue, skin color, body dimensions (physique), physical and physiological functions, quality of waste matter (stool, urine, sweat), sleep and wakefulness, impressibility speed and psychic function (5, 6). Although there is no identical definition in conventional medicine, some similarities have been noticed, such as in the result of the study by Rezadoost et al. which showed people from opposite Mizaj groups (warm-dry vs cold-wet) have significant difference in their proteomics (7). According to the result of this study, it can be theorized that Mizaj is a demonstration of genome. According to PM theories, determining temperament can also help identify other psychological, and physical characteristics of a person because each temperament is associated with certain types of characteristics, for example, if a person's temperament is warm, it can be assumed that she or he usually has more physical activity and wider social relationships and vice versa (6). So far, some studies have revealed the existence of some of these associations. For example, Salmannejad's study showed that there is a relationship between happiness and Mizaj. Indeed, the frequency of happy individuals was more in participants with warm Mizaj in this study (5). Parvizi et al.‘s study also showed the relation between BMI and Mizaj (8). Moreover, some studies revealed the relationship between types of Mizaj and diseases. As an instance, evaluating the patients with multiple sclerosis showed their Mizaj was inclined to coldness significantly (9). According to this point of view, the chance of contracting diseases can be predictable (9, 10). Other factor related to the Mizaj is the quality of functions of various organs. According to this theory, the abilities of the brain, such as the five senses, are better in people with a warm Mizaj than in people with a cold Mizaj. Memory as one of the functions related to the brain is considered to be usually stronger in warm or dry Mizaj and weaker in cold or wet Mizaj too (3, 5). As mentioned before, efforts have been made to evaluate the Mizaj effects on physical and mental ability (6, 11) but no study has yet been conducted on relationship between Mizaj and memory. So, the aim of this study was to assess the correlation between Mizaj and memory score as a scientific appraisal of PM concepts. Besides, if this relationship was proven, memory score can be used as one of the criteria for determining of Mizaj. 

## Methods

This study was a cross-sectional (descriptive correlational) study and the target population was the 18 to 38 years old students of both sexes and different ethnicities of Babol University of Medical Sciences, Babol, Iran. The convenience sampling was conducted by poster advertisement in university dormitories, as well as visiting students in their room and explaining the goals and method of the study and asking them to participate in the project. Inclusion criteria included all 18-38 age old students of Babol University of Medical Sciences. Definitive exclusion criteria were known psychiatric illnesses, history of any chronic disease such as hypothyroidism and diabetes, consumption of any kind of medications routinely, and history of using this test in the past. The volunteers were asked about temporary stress and anxiety, sleeplessness and tiredness which can affect memory and if they had a report about each of them, they were asked to return one week later if they want.

**Measurements and Statistical Analysis**

**Mizaj assessment:** Mojahedi’s Mizaj questionnaire (MMQ) which included 10 questions was used for determining the whole Mizaj of participants.

This questionnaire was designed and validated by Mojahedi et al. in 2014. Questionnaire questions include hand coldness or warmness, palm size, speed of affecting by heat and cold, speed of sentences expression, strength and tone of voice, speed of body movements, obesity and thinness, and softness and dryness of the skin. Answers are in likert form of 3 responses. The first 8 questions measured warmness-coldness (total score> 18 = warm, 15 - 18 is temperate in warmness-coldness and < 15 = cold) the last 2 questions measured wetness-dryness (total score> 4 = dry, 4 is temperate in wetness-dryness and < 4 = wet). The sensitivity and specificity of the questionnaire based on selected cutoff points were 65% and 93% for warm group, 52% and 97% for cold group, 53% and 67% for dry group and finally, 53% and 76% for wet group (6).

**Memory assessment:** For this purpose, Wechsler Memory Scale III (WMS III), which is one of the most complete, practical and available tests, was used. The physical Persian version of this test was purchased. It has 11 primary subsets and 7 optional subsets (12), sum of these subsets resulted in making 8 indexes. 

These 11 subsets include Logical Memory I, Verbal Paired Associates I, Faces I, Family Pictures I, Logical Memory II, Verbal Paired Associates II, Faces II, Family Pictures II, Logical Memory Recognition, Verbal Paired Associates Recognition, Letter-Number Sequencing, Spatial Span, each of them has particular scores.

The sum of scores ranges from 0 to 496 and called pure memory score that is convert to Memory Quotient (MQ) using a standard table of matching numbers. Performing the test completely takes 45 minutes averagely for each participant. In one usual classification, four types of memory are extracted from the 8 indexes which are auditory memory, visual memory, general memory and working memory. The range of scores are 0-155, 0- 244, 0- 176, 0-53, respectively (12).

**Sample size:** The main aim of the study was to assess the relationship between warm-cold Mizaj and memory score. So, considering the previous studies in this field, sample study considered 30 participants with warm Mizaj and 30 participants with cold Mizaj.

**Study protocol:** At the first step, participants were asked to complete MMQ. The sum of the scores on the form was calculated immediately, and if the person had a cold or warm (no moderate Mizaj) Mizaj based on that score, they were asked to take the Wechsler test.

**Statistical analysis:** The collected data were analyzed by SPSS Version 22 and the chi square (x2) and t-test were run. The p-value 0.05 was considered as significant difference in this study.

**Ethical consideration:** Ethical approval was received from Babol University of Medical Sciences (ID: IR.MUBABOL.HRI.REC.1398.161). After describing the aim and steps of the study and assuring the participants that their test results would be kept confidential, they were asked to sign the form of informed consent.

## Results

This study was conducted from August 2020 to October 2020 and 80 volunteers completed MMQ, until 30 participants with warm and 30 with cold were achieved. Forty -two of the participants were females and 18 were males. Their average age was 23.6 (21-27 years). Considering the sum score of the last two question of MMQ, 20 (57.2%) participants had wet Mizaj and 15 (42.8%) participants had dry Mizaj and the rest had a mild temperament on wet and dry domain. The average of MQ was 122.1 ± 5.7, the minimum score was 104.6 and the maximum was 126. There is no significant difference between MQ score of males and females (table 1).

The average of MQ in warm Mizaj was 125.46 ± 1.2 and in cold Mizaj was 118.79 ± 6.5. The difference between the two groups was statistically significant (p< 0.001). The average of MQ in dry Mizaj was 124.16 ± 2.67 and in wet Mizaj was 118.40 ± 7.64. The difference between two groups was statistically significant (P= 0.005) There was a significant relationship between warm/cold Mizaj with all subtypes of memory (table 2) as well as between dry/wet Mizaj with all subtypes of memory except working memory (table 3). It means students with warm Mizaj had better memory score than students with cold Mizaj and students with dry Mizaj had better memory score than students with wet Mizaj. 

**Table 1:** The average of Memory Quotient (MQ) in males and females

**p-value**	**SD**	**Average MQ**	**Gender**
0.24	5.5	123.46	**Male**
	5.7	121.56	**Female**

**Table 2:** The average of Memory Quotient (MQ) in Warm/Cold Mizaj in the subtypes of memory

**Subtypes of Memory**	**Type of Mizaj** **warm/cold**	**MQ average**	**SD**	**P-value**
**Auditory memory**	**warm**	141.8	2.6	<0.001
	**cold**	132.4	7.4	
**Visual memory**	**warm**	213.3	2.8	<0.001
	**cold**	206.3	6.8	
**General memory**	**warm**	169.7	3.2	<0.001
	**cold**	164.4	4.4	
**Working memory**	**warm**	39.6	0.8	0.002
	**cold**	38.8	1.1	

**Table 3:** The average of Memory Quotient (MQ) in Dry/Wet Mizaj in the subtypes of memory

**Subtypes of Memory**	**Type of Mizaj** **(Dry/Wet)**	**MQ average**	**SD**	**P-value**
**Auditory memory**	**Dry**	140.4	5.2	0.002
	**Wet**	132.7	8.4	
**Visual memory**	**Dry**	212.5	3.6	0.003
	**Wet**	206.1	7.8	
**General memory**	**Dry**	168.8	4.8	0.012
	**Wet**	164.5	4.6	
**Working memory**	**Dry**	39.1	1.0	0.44
	**Wet**	38.8	1.1	

## Discussion

The results showed there are significant relationships between memory score and warm/cold Mizaj and dry /wet Mizaj. Indeed, memory score was higher in warm Mizaj and in dry Mizaj. This association was also detected between the subtypes of memory and Mizaj expect between working memory and dry/wet Mizaj. These results are in accordance with theories in PM which indicate people with warm Mizaj and dry Mizaj have better memory and people with cold Mizaj and wet Mizaj have weaker memory and are more at risk of memory dysfunction (6, 9).

There is no research for evaluating memory based on Mizaj but the relationship between types of Mizaj and some physiological, physical and psychological aspects of human health has been evaluated in some other studies. One study by Banaee et al. showed there is a relationship between types of Mizaj and academic motivation among the students of nursing and midwifery (13). The results of this study showed the significant more academic motivation in students with hot and dry temperament. Motivation is one of the most important psychological concepts in education and is related to several variables such as cognitive functions such as verbal memory (14, 15). 

Another study by Salman-nejad et al. showed the significant relationship between warm Mizaj and happiness using MMQ and Oxford Happiness Questionnaire in students of Tehran universities (5). According to some researches, positive mood could enhance working and autobiographical memory capacity (16, 17). Indeed in these studies, the relationship between related factors to memory and Mizaj has been revealed too. The relationship between other brain’s function, speaking speed, and warm Mizaj also has been shown in study by Noor et al. They concluded that evaluating the voice can be considered as a parameter in Mizaj identification (18).

 It seems that according to studies which have been conducted till now, warm Mizaj could enhance brain activities such as memory, motivation, happiness and speaking speed. 

According to this viewpoint, PM scholars recommended herbal medicines or diet with warm Mizaj such as prunus amygdalus, corylus avellana L., crocus sativus L., cuminum cyminum Linn, foeniculum vulgare L. and melissa officinalis L. as brain functions boosters which also can improve memory. Nowadays, their probable phytochemical mechanisms are known (19, 20). 

However, in some studies, despite the identification of the relationship between the considered characteristic and Mizaj, this relationship does not completely match the descriptions in PM sources. As an instance according to a study by Tavoosi and Mazaheri, there is a relationship between personality types and Mizaj among the medical students in Isfahan University of Medical Sciences but some charectristices such as directedness, cooperativeness, and self-transcendence had the least prevalence among the persons with warm and dry Mizaj (21) contrary to the PM theory, which considers the lack of these charectristices to be due to cold and dry Mizaj (6).

There are some theories to describe Mizaj according to new biological mechanisms which are supported by some evidence such as the theory that claims sympathetic nervous system activity and thyroid function are effective factors in Mizaj forming and they have been revealed to have few activities in people with cold Mizaj (22). It seems there may be a relationship between these factors and brain’s functions such as memory too (23). Among them, the relationship between memory and thyroxine level was investigated the most (24). However, a great number of studies are needed to illustrate the relationship between memory, Mizaj and these factors and their exact mechanisms.

There is a notable point that according to PM references, the relationship between memory and brain Mizaj is stronger than between memory and whole body Mizaj. Although a questionnaire and a checklist has been designed for brain Mizaj identification recently (25), considering the lack of that at the time of performing this study, we applied MMQ as the whole body Mizaj questionnaire inevitably. So, this can be considered as an important limitation for our study and as a suggestion for future studies. The small sample size also can be mentioned as another limitation. Moreover, recruitment of students in certain age range which reduces the impacts of age changes on memory is an advantage for our study but while all of them are medical sciences students who are usually considered to have good brain abilities, it may have affected the results. Hence, performing studies with larger and diverse sample size is recommended too. 

This study was the first step of evaluating the relationship between memory and Mizaj. The results provided some evidence to use memory as an index to differentiate cold and warm Mizaj and wet and dry Mizaj in related questionnaire. According to this result, more studies in finding the causality also can be recommended. If the relationship is confirmed by more studies, it may be possible to predict individual’s susceptibility to memory weakness according to them.
